# Synergistic Phytotoxic Effects of Culmorin and Trichothecene Mycotoxins

**DOI:** 10.3390/toxins11100555

**Published:** 2019-09-20

**Authors:** Rebecca Wipfler, Susan P. McCormick, Robert H. Proctor, Jennifer M. Teresi, Guixia Hao, Todd J. Ward, Nancy J. Alexander, Martha M. Vaughan

**Affiliations:** United States Department of Agriculture, Agricultural Research Services, National Center for Agricultural Utilization Research, Peoria, IL 61604, USA; rlwipfler@gmail.com (R.W.); susan.mccormick@usda.gov (S.P.M.); Robert.Proctor@usda.gov (R.H.P.); Jennifer.Teresi@usda.gov (J.M.T.); Guixia.Hao@usda.gov (G.H.); Todd.Ward@usda.gov (T.J.W.); vernan22@comcast.net (N.J.A.)

**Keywords:** culmorin, trichothecene, deoxynivalenol, nivalenol, mycotoxins, synergistic phytotoxicity, *Fusarium*, cereal crops

## Abstract

Species of the fungus *Fusarium* cause Fusarium head blight (FHB) of cereal crops and contaminate grain with sesquiterpenoid mycotoxins, including culmorin (CUL) and trichothecenes. While the phytotoxicity of trichothecenes, such as deoxynivalenol (DON), and their role in virulence are well characterized, less is known about the phytotoxicity of CUL and its role in the development of FHB. Herein, we evaluated the phytotoxic effects of purified CUL and CUL-trichothecene mixtures using *Chlamydomonas reinhardtii* growth and *Triticum aestivum* (wheat) root elongation assays. By itself, CUL did not affect growth in either system. However, mixtures of CUL with DON, 3-acetyldeoxynivalenol, 15-acetyldeoxynivalenol, or NX-3, but not with nivalenol, inhibited growth in a synergistic manner. Synergistic phytotoxic effects of CUL and DON were also observed on multiple plant varieties and species. The severity of wheat FHB caused by 15 isolates of *Fusarium graminearum* was negatively correlated with the CUL/DON ratio, but positively correlated with the sum of both CUL and DON. Additionally, during the first week of infection, CUL biosynthetic genes were more highly expressed than the *TRI5* trichothecene biosynthetic gene. Furthermore, genomic analysis of *Fusarium* species revealed that CUL and trichothecene biosynthetic genes consistently co-occur among species closely related to *F. graminearum*.

## 1. Introduction

Fusarium head blight (FHB) is one of the most destructive diseases of wheat and other cereal crops worldwide. During infection, *Fusarium* species, such as *Fusarium graminearum,* produce an array of secondary metabolites, including mycotoxins, that can pose serious risks to plant, animal, and human health [1]. Trichothecenes are among the most problematic *Fusarium* mycotoxins because they frequently contaminate small-grain cereals, which raises food safety concerns and requires regulation [2,3]. Trichothecenes are sesquiterpenoid metabolites. Biosynthesis is initiated when farnesyl diphosphate (FPP), the common precursor of all sesquiterpenes, is converted by the terpene synthase Tri5 into the hydrocarbon volatile trichodiene. Trichodiene then undergoes a series of enzyme-catalyzed modifications to form trichothecenes such as nivalenol (NIV), deoxynivalenol (DON) and its acetylated derivatives 3-acetyldeoxynivalenol (3-ADON) and 15-acetyldeoxynivalenol (15-ADON), diacetoxyscirpenol, T-2 toxin, and NX-2 [1,4]. *Fusarium graminearum* predominantly contaminates grain with type B trichothecenes including DON and NIV, or the type A trichothecene NX-3. The acetylated trichothecenes produced by *F. graminearum* in culture, e.g., 3-ADON, 15-ADON, 4-ANIV, 4,15-diANIV, and NX-2, are mostly converted to their deacetylated forms, DON, NIV, and NX-3, in planta. Evidence of the phytotoxicity of these structural analogs has been described in numerous studies [5,6,7]. DON, in particular, is a potent inhibitor of eukaryotic protein synthesis and serves as a virulence factor, enabling *Fusarium* to overcome natural plant defences and spread throughout the wheat spike [8,9]. Considerable research has been devoted to understanding the role of trichothecenes in host colonization and pathogen aggressiveness; however, relatively little is known about the potential synergistic interactions with other *Fusarium* secondary metabolites [10,11,12].

Culmorin (CUL) is a tricyclic sesquiterpene diol derived from activity of the terpene synthase Clm1, which converts FPP into longiborneol [13], and the cytochrome P450 Clm2, which catalyzes the hydroxylation of longiborneol to CUL [14]. Naturally contaminated grain samples have relatively high concentrations of CUL, and levels are typically positively correlated with the amount of DON [2,15]. Thus, CUL is considered to be an “emerging mycotoxin” [2,16]. While CUL does not appear to affect insects or animals by itself, the co-occurrence of CUL and DON can have a synergistic effect on toxicity [17,18]. Recent findings indicate that CUL can supress the activity of uridine diphosphate glucosyltransferases (UGTs) that catalyze the glycosylation of DON into the less toxic DON 3-O-glucose (D3G) [19]. UGT enzymes are found in all living organisms, and inhibitors of such enzymes may function across a broad taxonomic range as CUL has been demonstrated to inhibit UGTs from humans and rice [19] (and unpublished work referenced therein).

Metabolism of DON to D3G has been identified in most cereal crops, including *Triticum aestivum* (wheat), *Hordeum vulgare* (barley), and *Zea mays* (maize/corn) [2,20,21,22]. In wheat, FHB resistance to the spread of disease along the spike (also known as type II resistance) is closely correlated with the metabolism of DON into D3G [23,24,25]. UGTs capable of detoxifying DON via glycosylation have been identified in barley, rice, and wheat [22,26,27,28,29]. Overexpression of these UGTs in heterologous systems have demonstrated functionality in disease resistance. For example, overexpression of a barley UGT in wheat resulted in enhanced FHB resistance [30]. Similarly, expression of a wheat UGT (Traes_2BS_14CA35D5D) in *Brachypodium distachyon* increased FHB resistance and root tolerance to DON [26].

The role of CUL in *Fusarium* infection of plants is not well characterized. In contrast to insects and animals, wheat coleoptile tissues have been reported to be sensitive to CUL [5]. Comparisons between disease progression of CUL-producing *F. graminearum* strains and CUL non-producing mutant strains disrupted in CUL biosynthesis have yielded conflicting and inconclusive results. Gardiner et al. [31] reported that disruption of *CLM1* (FGSG_10397) or *CLM2* (FGSG_00007, also known as FGSG_17598) resulted in increased *TRI5* transcription, DON production, and aggressiveness in wheat. In contrast, Bahadoor et al. [14] reported that the disruption of *CLM2* did not affect the transcription of trichothecene biosynthetic genes, and mutant strains had reduced virulence on wheat in comparison to the wild-type or add-back strains. However, the control transformant strain also displayed a reduction in virulence and, thus, only one of the two *CLM2* mutants evaluated was significantly less virulent than the transformed control [14]. Similarly, inconsistent results between independent *CLM1* mutant strains and transformant controls were observed by Alexander and Vaughan (unpublished). Additionally, the disruption of *CLM2* led to the production of five novel metabolites: 3-hydroxylongiborneol, 5-hydroxylongiborneol, 12-hydroxylongiborneol, 15-hydroxylongiborneol, and 11-epi-acetylculmorin [14]. A drawback to assessing virulence phenotypes of mutant strains in which a secondary metabolite pathway has been blocked is that such mutations can be pleiotropic, as biosynthetic pathways other than the target pathway can be affected. These unintended changes in the metabolically engineered *Fusarium* strains likely confounded disease assay results and made it difficult to determine the role of CUL in host infection and colonization. Similar challenges to manipulating terpenoid biosynthetic pathways and understanding the regulatory complexity involved have previously been reviewed [32].

As the basis for this study, we hypothesized that the co-ocurrence of CUL and trichothecenes has synergistic phytotoxic effects. To overcome the challenges posed by the pleotropic effects of CUL-nonproducing mutants, we evaluated the direct phytotoxic effects of purified CUL by itself and in mixtures with trichothecenes. The phytotoxicity of the metabolites was initially assessed using the unicellular plant model system *Chlamydomonas reinhardtii* [7]. However, since plant roots are also sensitive to DON [26,33], we further assessed the effect of CUL by itself and CUL-trichothecene mixtures on root growth in wheat, barley and corn. Since mixtures consisting of higher levels of CUL than DON resulted in increased phytotoxicity, we further hypothesized that FHB severity would be positively correlated with the ratio of CUL/DON produced by the pathogen. Therefore, we evaluated the relationship between the CUL/DON ratio and FHB progression in wheat and compared the expression of CUL and DON biosynthetic genes during early stages of infection. Given the potential fitness benefits associated with co-production of DON and CUL, we conducted a genomic analysis of selected species of *Fusarium* to determine if there was an evolutionary association between the biosynthetic pathways of trichothecenes and CUL.

## 2. Results

### 2.1. Effects of CUL and Trichothecenes on C. reinhardtii Growth

**Figure 1:**
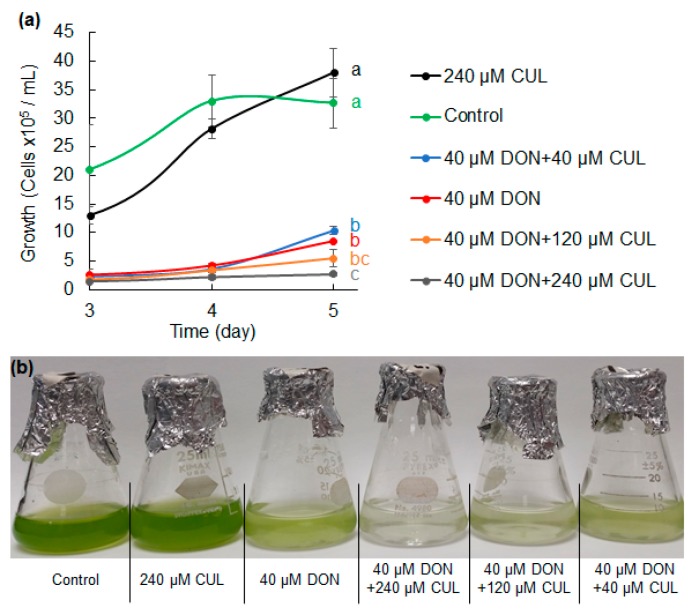
Synergistic phytotoxic effect of culmorin (CUL) and deoxynivalenol (DON) on *Chlamydomonas reinhardtii* growth. (**a**) Comparison of *C. reinhardtii* growth curves exposed to DON and CUL at various concentrations. Points represent average ± standard error of the mean (SEM) concentration of cells. Different letters indicate significant differences between mean (repeated measures (RM) ANOVA and Tukey–Kramer honestly significant difference (HSD), *p* < 0.03, *n* = 3). (**b**) Representative image of phytotoxicity assay using *Chlamydomonas reinhardtii* as a model.

To assess the phytotoxicity of CUL and CUL-trichothecene mixtures, the growth of *C. reinhardtii* cultures treated with the purified mycotoxins was evaluated over time. Treatment with 40 μM DON significantly reduced *C. reinhardtii* growth to approximately a fourth of the control, and this concentration was chosen for further experiments (Figure 1). Treatment with 40, 120, or 240 μM CUL did not significantly affect the growth of *C. reinhardtii*, and equimolar amounts (40 μM) of CUL and DON did not significantly alter *C. reinhardtii* growth in comparison to cultures treated with only DON. Mixtures of DON with three or six times more CUL further reduced *C. reinhardtii* growth in relation to the DON only treatment (Figure 1). However, this reduction was statistically significant only in the treatment with CUL at six times the amount of DON, in which *C. reinhardtii* growth was reduced to 68% of the DON only treatment (*p* = 0.02).

**Figure 2:**
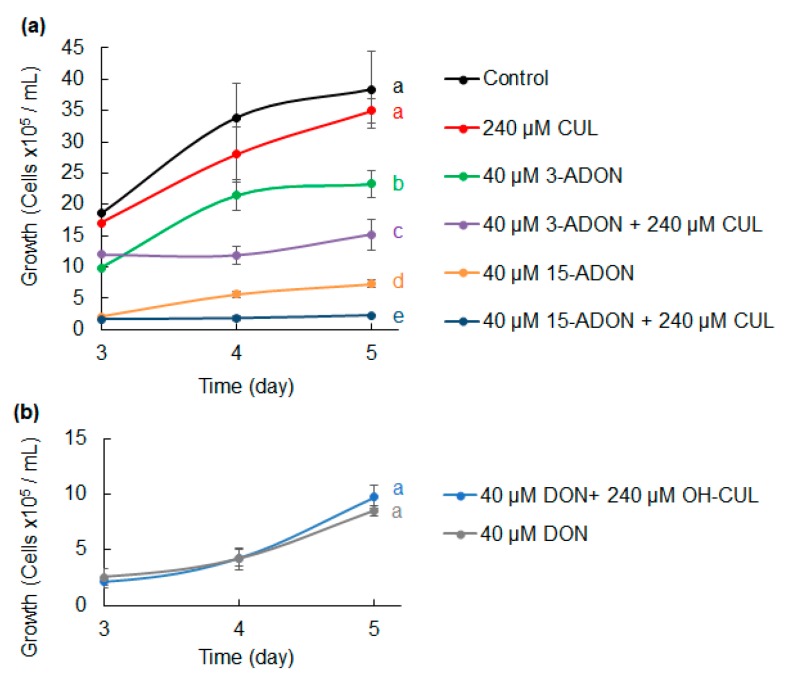
Combined effect of structural analogs of DON and CUL on *Chlamydomonas reinhardtii* growth. (**a**) Comparison of *C. reinhardtii* growth when not exposed (control) or exposed to CUL, 15-ADON, or 3-ADON, and various combinations of these metabolites, and (**b**) hydroxy-culmorin (OH-CUL) and DON. Different letters to the right of growth curves indicate significant differences between means (RM ANOVA and Tukey–Kramer HSD, *p* < 0.05, *n* = 3).

To determine if CUL had a similar synergistic effect with other trichothecene analogs produced by *F. graminearum* (i.e., 15-ADON and 3-ADON), growth in the presence of each analog by itself and in mixtures with CUL was assessed (Figure 2a). *C. reinhardtii* growth was significantly inhibited by 40 μM 15-ADON or 3-ADON in comparison to controls and treatment with 240 μM CUL (*p* < 0.01). Growth was inhibited further when the cultures were treated with mixtures of 240 μM CUL and 3-ADON or 15-ADON. In comparison to corresponding treatments with ADON compounds, a mixture of 40 μM 15-ADON and 240 μM CUL reduced growth by 71% (*p* = 0.008), and a mixture of 40 μM 3-ADON and 240 μM CUL reduced growth by 35% (*p* = 0.03).

Because the CUL derivative hydroxy-culmorin (OH-CUL) is frequently found as a co-contaminate with CUL in grain, we also evaluated whether OH-CUL had a similar negative effect with DON on growth (Figure 2b). However, the repeated measures analysis of variance indicated that the growth of *C. reinhardtii* cultures treated with a mixture of 40 μM DON and 240 μM OH-CUL did not significantly differ from those treated with just 40 μM DON.

### 2.2. Effects of CUL and Trichothecenes on the Growth of Wheat Roots

**Figure 3:**
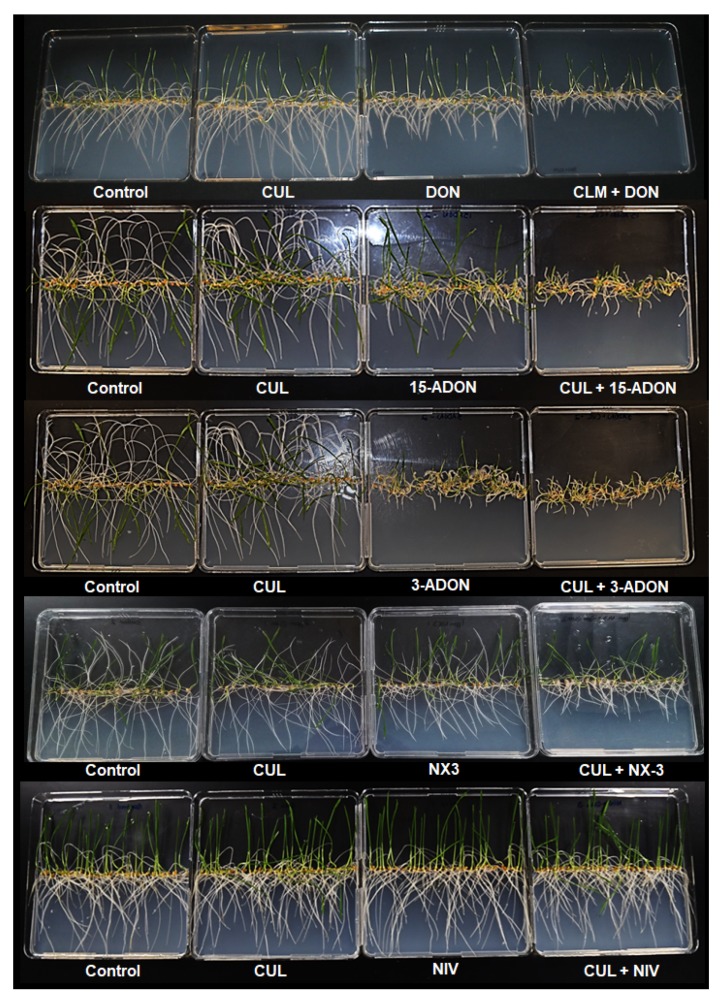
Images of root phytotoxicity assays on 1.5% water agar illustrating the effect of 2 ppm CUL and 2 ppm of other *Fusarium graminearum* sesquiterpenoid mycotoxins on wheat (variety ‘Norm’) root elongation.

**Figure 4:**
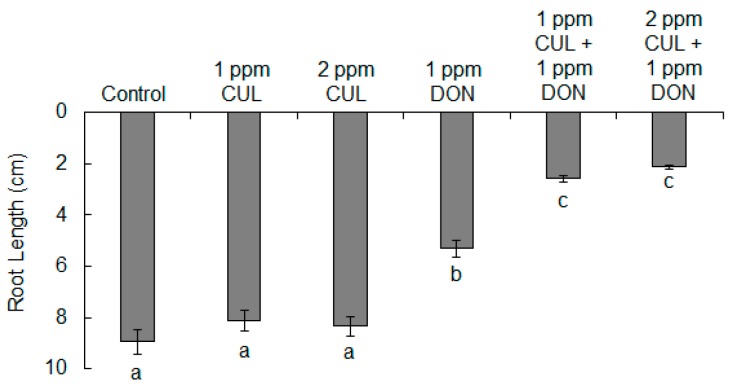
Effects of CUL and DON on wheat root elongation. Average root length ± standard error of the mean of ‘Norm’ wheat roots after five days of growth on 1.5% water agar containing the different mycotoxin treatments. Significantly different means are indicated by different letters below the bars according to ANOVA followed by Tukey–Kramer HSD (*n* = 90, *p* < 0.01).

The phytotoxicity of CUL and CUL-trichothecene mixtures on wheat was evaluated by assessing seedling root elongation after 5 days of growth on water agar containing the mycotoxins (Figure 3). Preliminary experiments determined that wheat roots were much more sensitive to DON than was *C. reinhardtii*. Approximately, 10-fold less DON, 3.4 µM (1 ppm), was sufficient to significantly reduce wheat (variety ‘Norm’) root growth by approximately 50% (Figure 4; *p* < 0.01). Consistent with the results from *C. reinhardtii* assays, treatment with CUL alone did not have any significant phototoxic effect on wheat root elongation. Nevertheless, the relative amount of CUL needed to result in a phytotoxic effect when combined with other trichothecenes was less in the wheat root assay as compared to the *C*. *reinhardtii* assay. Concentrations of 4.2 µM CUL (1 ppm; 1.2-fold in excess to DON) or 8.4 µM (2 ppm; 2.5-fold in excess to DON) with 3.4 µM DON (1 ppm) resulted in a significant synergistic inhibitory effect on wheat root elongation in comparison to treatment with 1 ppm DON alone (*p* < 0.01). Roots grown on media containing 2 ppm CUL and 1 ppm DON were about 50% shorter than roots grown on media with just 1 ppm DON, and were 80% shorter than the control (Figure 4). For further experiments, the concentrations of 2 ppm and 1 ppm were chosen for CUL and other sesquiterpenoids, respectively.

**Figure 5:**
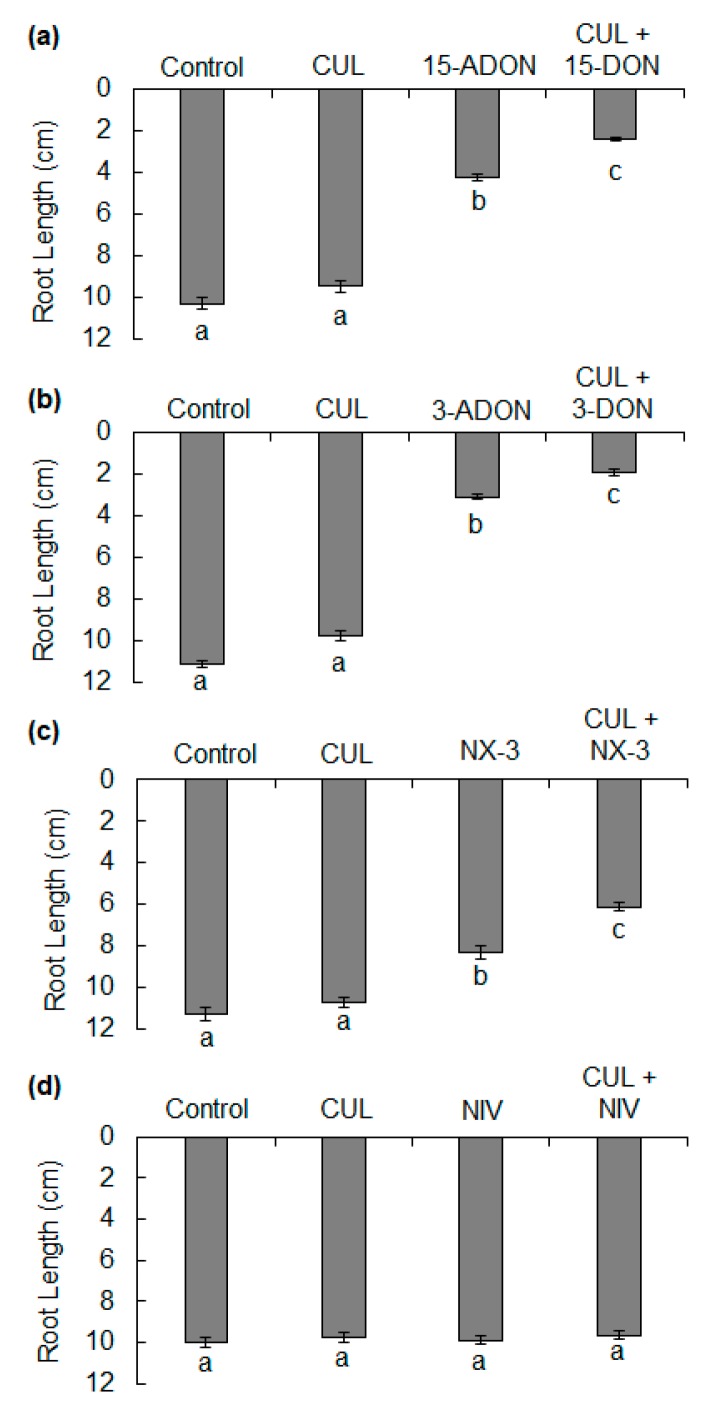
Effects of CUL and other structural analogs of DON on ‘Norm’ wheat root elongation. Average length of roots after five days of growth on 1.5% water agar containing combinations of 2 ppm CUL with (**a**) 1 ppm 15-DON, (**b**) 1 ppm 3-ADON, (**c**) 1 ppm NX-3 or (**d**) 1 ppm NIV. Data represent averages ± SEM from three independent experimental plates. Different letters below the bars indicate significant differences among treatments (ANOVA and Tukey–Kramer HSD, *n* = 90, *p* < 0.05).

The phytotoxicity of CUL with 15-ADON, 3-ADON, NX-3 or NIV was also evaluated (Figure 3 and Figure 5). Independently, 1 ppm 15-ADON, 3-ADON, or NX-3 significantly reduced root elongation in comparison to controls (Figure 5a–c). In combination with 2 ppm CUL, 15-ADON, 3-ADON, or NX-3 reduced root growth even further, resulting in 45%, 40%, and 32% reduction, respectively, in root length in comparison to the respective single trichothecene (*p* < 0.05).

**Figure 6:**
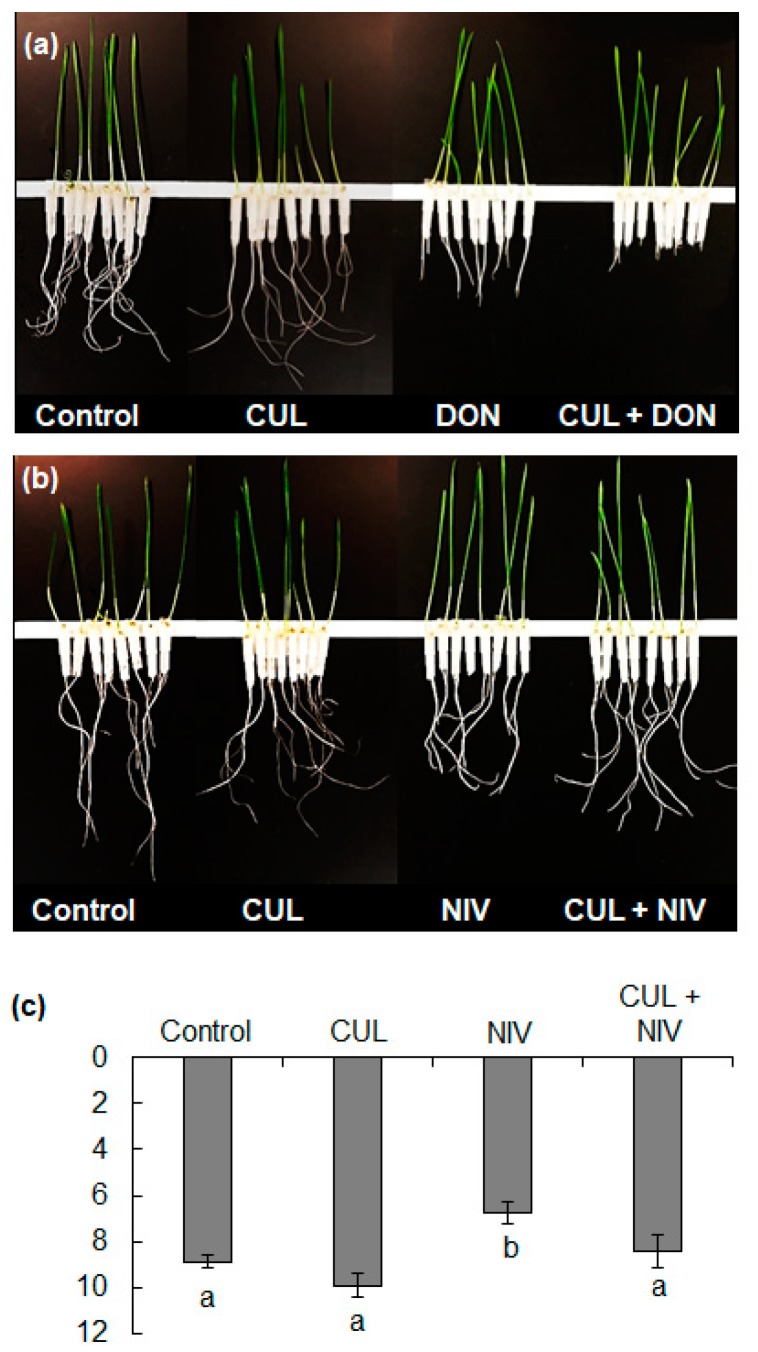
Images of the hydroponic root phytotoxicity assays, in which wheat (‘Norm’) seedlings were grown in purified water containing the different mycotoxins. (**a**) Image of a representative assay with 2 ppm CUL and 1 ppm DON. (**b**) Image of a representative assay with 2 ppm CUL and 1 ppm NIV. (**c**) Average length of roots after five days of growth in hydroponic culture containing 2 ppm CUL with 1 ppm NIV. Data represent averages ± SEM from three independent experimental replicates. Different letters below the bars indicate significant differences between treatments (ANOVA and Tukey–Kramer HSD, *n* = 24, *p* < 0.05).

In contrast, neither 1 ppm NIV nor 1 ppm NIV with 2 ppm CUL significantly reduced root elongation on water agar (Figure 5d). However, during these initial experiments, many roots were observed lifting from the agar, presumably to avoid direct contact with the mycotoxins. Since this avoidance behavior could have skewed the results, we conducted additional root growth experiments using hydroponic assays, where the root tissues could only grow in direct contact with liquid media containing the mycotoxins. The resulting inhibition of hydroponically grown roots treated with 1 ppm DON and 2 ppm CUL was similar to that observed in the water agar assay (Figure 6a). Additionally, in the hydroponic assay, 1 ppm NIV reduced root elongation by 23% in comparison to controls, but the combined treatment of 1 ppm NIV with 2 ppm CUL did not result in synergistic phytotoxicity (Figure 6b,c). Additionally, in the hydroponic assay, 1 ppm NIV reduced root elongation by 23% in comparison to controls, but the combined treatment of 1 ppm NIV with 2 ppm CUL did not result in statistically significant phytotoxicity (Figure 6b,c), suggesting that CUL helped to protect the roots from the phytotoxic effects of NIV (Figure 6c).

### 2.3. Synergistic Phytotoxicity of CUL and DON on Multiple Varieties of Wheat, Barley and Corn

**Figure 7:**
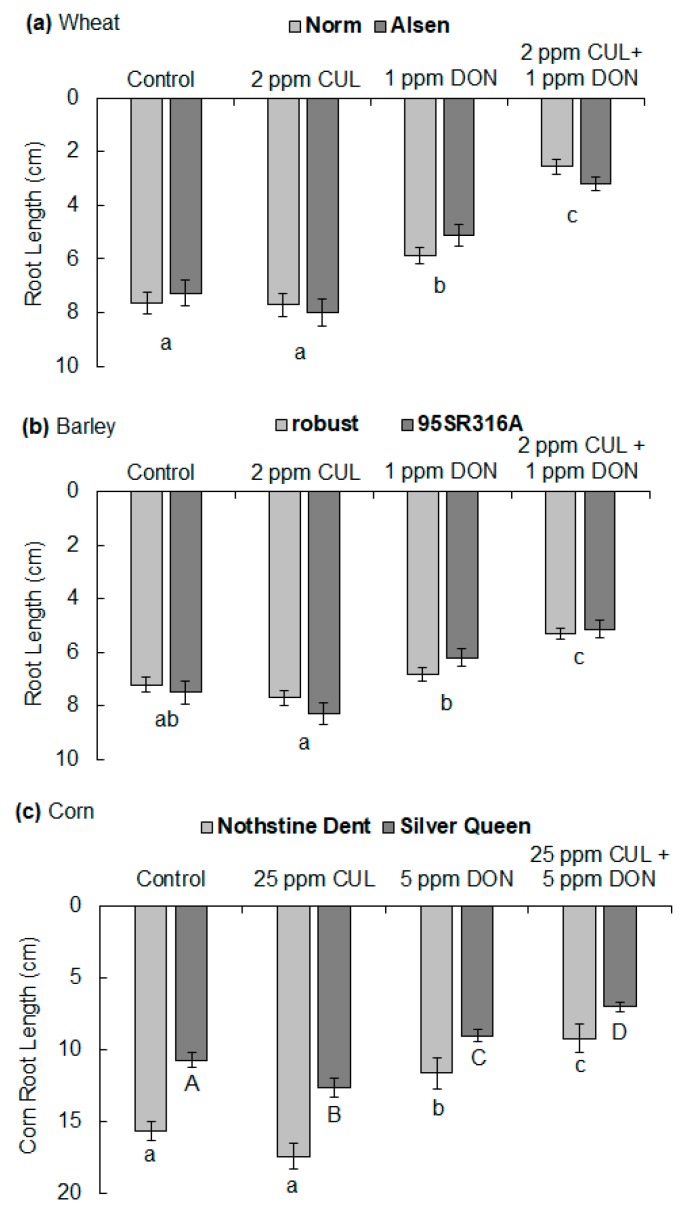
Effect of CUL and DON on the root elongation of different varieties and species of cereal crops. Data represent average (**a**) wheat, (**b**) barley, and (**c**) corn root length ± SEM after five days of growth on 1.5% water agar containing different sesquiterpenoid mycotoxin treatments. For (**a**) wheat and (**b**) barley, a 2 × 4 (variety × treatment) full factorial ANOVA determined that there was no significant difference between the two varieties evaluated. Therefore, the data were further analyzed together, and differences between treatments, indicated by letters below the bars, were assigned according to post hoc Tukey–Kramer HSD (*n* = 60, *p* < 0.01). Root elongation of the (**c**) corn varieties significantly differed according to the 2 × 4 full factorial ANOVA, and thus they were further analyzed independently. Corn roots were also more tolerant to DON, and higher concentrations of DON and CUL were used to distinguish significant differences between the treatments (ANOVA followed by Tukey–Kramer HSD (*n* = 30, *p* < 0.05)).

To determine if the synergistic phytotoxicity of CUL with DON was host-specific with regard to variety and/or species, water agar root growth assays were conducted with the moderately resistant spring wheat variety ‘Alsen’ and two varieties of barley and corn of variable resistance levels. When grown on water agar containing 2 ppm CUL and 1 ppm DON, ‘Norm’ and ‘Alsen’ seedlings had on average 56% and 38% shorter roots, respectively, in comparison to corresponding roots grown on water agar containing only 1 ppm DON (Figure 7a; *p* < 0.001). However, a full factorial analysis of variance (ANOVA) determined that wheat variety was not a significant factor contributing to differences between treatment means. Likewise, no significant differences were observed between the two barley varieties, ‘Robust’ and ‘95SR316A’, that were evaluated. Barley roots were more tolerant of DON, and their root length was not significantly inhibited by 1 ppm DON in comparison to controls. However, a mixture of 1 ppm DON and 2 ppm CUL significantly inhibited root elongation of both barley varieties by approximately 20% (Figure 7b; *p* = 0.04). Variety was a significant factor contributing to the root elongation of the two corn varieties ‘Nothstine Dent’ and ‘Silver Queen’ (*p* < 0.0001), and therefore differences between treatments were analyzed separately for each variety. When exposed to 1 ppm DON, neither corn variety displayed a significant reduction in root length, and only ‘Silver Queen’ displayed significant reduction in root length when exposed to the combination of 1 ppm DON and 2 ppm CUL (data not shown). However, at 5 ppm DON, both corn varieties showed a significant reduction in root growth, and the synergistic phytotoxicity of 25 ppm CUL with 5 ppm DON further inhibited root growth by 20% for ‘Nothstine Dent’ and 22% for ‘Silver Queen’ (Figure 7c; *p* < 0.001).

### 2.4. Relationship between FHB and the Ratio of CUL/DON

**Figure 8:**
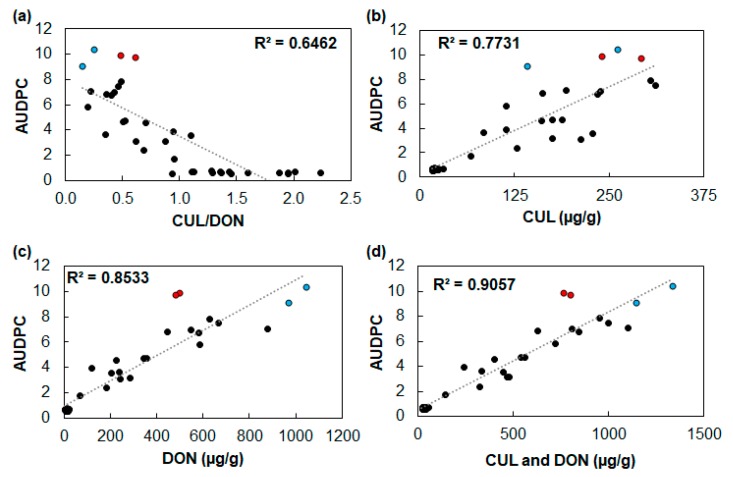
Relationship between the area under the disease progress curve (AUDPC) and the CUL to DON ratio. Flowering ‘Alsen’ wheat heads were inoculated with 15 individual *F. graminearum* strains, disease progression over time was monitored, and then the AUDPC was plotted against the quantified endpoint (day 21) concentration of (**a**) CUL/DON, (**b**) CUL, (**c**) DON, and (**d**) the sum of CUL and DON. Each point represents data from a single wheat head inoculated with a single strain, and three independent heads were inoculated per strain. The linear trendlines and coefficients of determination were estimated for each graph. Red and blue points are discussed in the text.

Since the combination of CUL and DON generally had a synergistic phytotoxic effect that was most apparent when CUL was present at a higher concentration than DON, we hypothesized that disease would be positively correlated with the ratio of CUL/DON produced by the pathogen. Therefore, we point-inoculated ‘Alsen’ wheat spikes with 15 individual *F. graminearum* strains (i.e., one strain per spike), assessed disease progression over time, and then quantified the amount of the sesquiterpenoid mycotoxins DON and CUL from harvested spikes. Each head was inoculated with a single strain, and each strain was inoculated into three independent wheat heads, resulting in a total of 45 heads that were analyzed (Figure 8a). However, contrary to our hypothesis, there was a modest negative correlation between the area under the disease progress curve (AUDPC) and CUL/DON, (y = −4.49x + 7.96; *R^2^* = 0.65; Figure 8a). Only 14% of the heads had a CUL/DON ratio greater than 1.5, and all of these had very low AUDPC values. Approximately 58% of the wheat heads had a CUL/DON ratio of less than 1. Among these were the four wheat heads with the highest AUDPC values, and their CUL/DON values ranged from 0.15 to 0.6, indicating that heads with the highest AUDPC values had more DON than CUL contamination.

Nevertheless, AUDPC was positively correlated with the concentration of CUL (y = 0.0286x + 0.2555; *R^2^* = 0.77) and DON (y = 0.01x + 0.9612; *R^2^* = 0.85) (Figure 8b,c). In addition, the sum of DON and CUL was most predictive of the AUDPC (y = 0.0078x + 0.5128; *R²* = 0.91; Figure 8d). Interestingly, among the four spikes with the highest AUDPC values (Figure 8), two spikes (indicated in red) have approximately half the amount of DON in comparison to the other two spikes (indicated in blue), and yet they caused similar amounts of disease. A key difference between the spikes indicated in red is that they have a higher relative CUL/DON ratio (0.5 and 0.6) in comparison to those indicated in blue (0.14 and 0.2).

### 2.5. Transcriptional Analysis of CUL and Trichothecene Biosynthetic Genes

**Figure 9:**
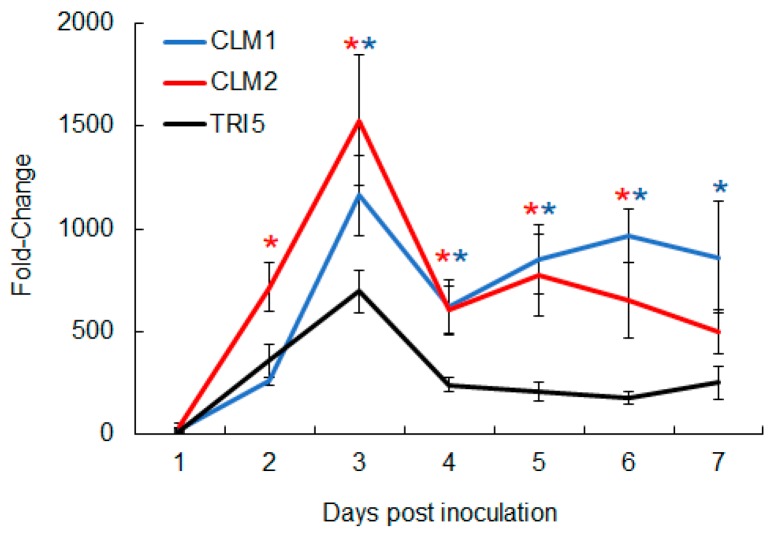
Transcriptional analysis of CUL and DON biosynthetic genes during the first 7 days post inoculation. cDNA was synthesized from wheat heads collected following dip inoculation. Six heads were collected at each time point from day 1 to day 7, and two heads were combined as one sample for RNA isolation. Gene expression was determined by RT-qPCR. Fungal β-tubulin was amplified as an internal control for transcript normalization. Fold changes of gene expression were relative to *F. graminearum* PH-1 axenic culture, which was grown on a V8 plate for seven days. Error bars represent SEM, and colored asterisks indicate which means are significantly different from *TRI5*, which was set as the control in Dunnett’s test (*n* = 3, *p* < 0.05).

Since mycotoxin quantification was conducted at the endpoint of the disease progression analysis (day 21), the potential for differences in the timing of CUL and DON production was evaluated by comparing the expression of biosynthetic genes during the first week post inoculation. This early timeframe was chosen for gene expression analyses because previous studies have shown that the expression of most *TRI* genes can be observed by two days and peaks at three to four days after inoculation [34]. Overall, the biosynthetic genes of both DON and CUL followed a similar trend and peaked at 3 days post inoculation. However, significant differences could be observed as early as two days post inoculation (Figure 9). While transcription of the sesquiterpene synthase genes *TRI5* and *CLM1* at day 2 were approximately the same (*p* > 0.05), transcription of the cytochrome P450 gene *CLM2* was two-fold greater (*p* = 0.02). For the remainder of the 7-day time course of study, transcript levels of *CLM1* and *CLM2* remained higher than *TRI5*. This difference was significant (*p* < 0.01) for all days, with the exception of day 7, at which point *CLM2* transcript levels began to decline and were not significantly different from those of Tri5 (*p* > 0.05). Nevertheless, based on the transcriptional analysis, it is possible that CUL production was greater than DON production during the first week of infection.

### 2.6. Distribution of CUL and Trichothecene Biosynthetic Genes in Selected Species of Fusarium

**Figure 10:**
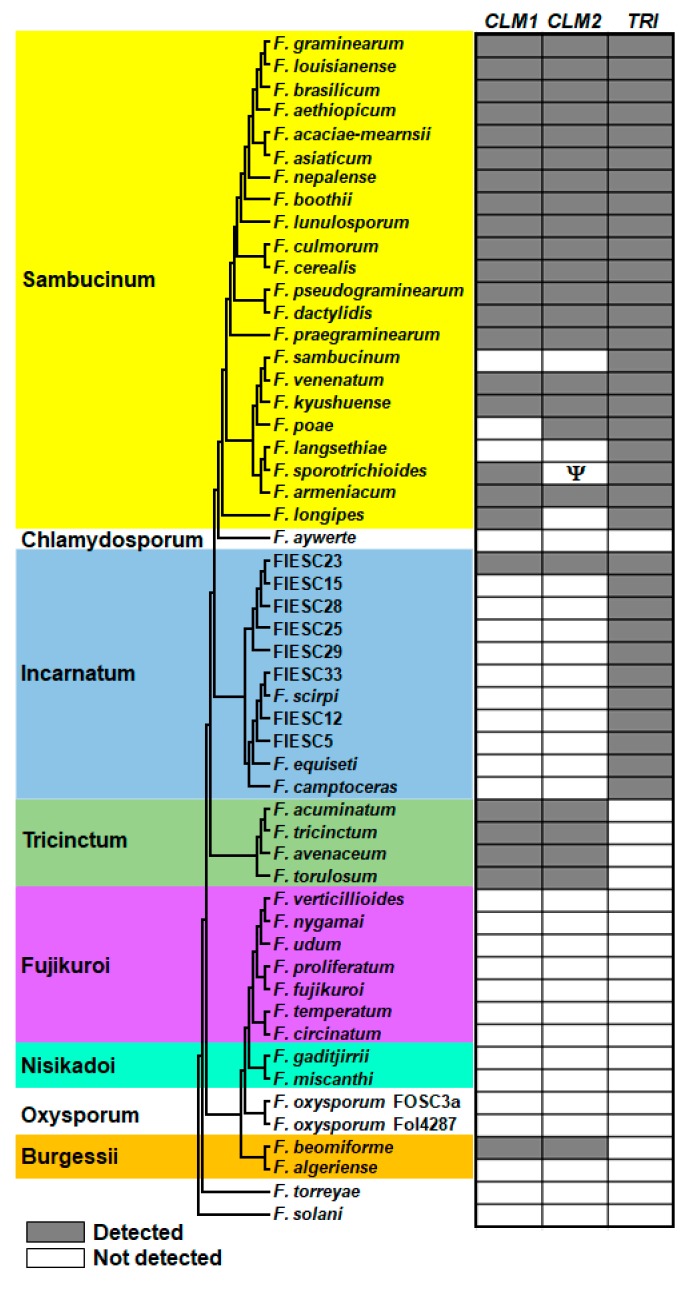
Distribution of culmorin (*CLM1* and *CLM2*) and trichothecene biosynthetic genes (*TRI*) in selected species of *Fusarium*. The tree on the left was inferred by maximum likelihood analysis of the concatenated alignment of 11 primary-metabolism genes. Species complexes are demarcated by colored boxes and are labeled using the species name after which each complex is named (e.g., Nisikadoi indicates the *F. nisikadoi* species complex) [35]. The columns on the right indicate the presence (gray) and absence (white) of biosynthetic genes in a genome sequence for a given species based on BLASTn analysis using orthologs of *F. graminearum* strain PH-1 genes as query sequences. The presence and absence of *TRI* genes were determined using selected genes as query sequences (*TRI1*, *TRI3*, *TRI4*, *TRI5*, *TRI6*, *TRI8*, *TRI10*, *TRI11*, *TRI12*, *TRI13* and *TRI14*). The Greek letter Ψ indicates a pseudogene; *F. sporotrichioides CLM2* has an internal stop codon near the middle of the coding region. Branch support values (bootstrap and internode certainty values) have been removed from the tree shown here but can be found in Figure S2. Strain numbers and genome sequence accessions for the species are shown in Table S1.

Since trichothecene-producing fusaria likely benefit from the production of CUL and DON, we hypothesized that genes from the two biosynthetic pathways would frequently co-occur. Therefore, we used BLAST analysis to assess the distribution of the two previously described CUL biosynthetic genes, *CLM1* and *CLM2* [13,14,34], in genome sequences of selected *Fusarium* species, including members of the *F. sambucinum* and *F. incarnatum*-*equiseti* species complexes (hereafter the Sambucinum and Incarnatum complexes, respectively). These complexes are multi-species lineages of *Fusarium* that have trichothecene biosynthetic genes, and trichothecene production has been confirmed in many of the species [35,36,37]. *CLM1* and *CLM2* occurred widely among members of the Sambucinum complex, which includes *F. graminearum*. Among the members of the Sambucinum complex that were examined, the genes occurred uniformly among species more closely related to *F. graminearum*, and sporadically among species more distantly related to *F. graminearum* (Figure 10). Among the genomes for the 11 species of the Incarnatum complex that were examined, homologs of both *CLM1* and *CLM2* occurred in only one species, the phylogenetically defined species FIESC 23. Homologs of *CLM1* and *CLM2* were also detected in four members of the *F. trincinctum* species complex (Tricinctum complex) and in *F. beomiforme*, which is a member of the *F. burgessii* species complex, but not in any of the other species examined. The *CLM1* and *CLM2* homologs in FIESC 23, *F. beomiforme* and the Tricinctum complex were distantly related to the homologs in the Sambucinum complex (Figure S1). Given these relatively distant relationships, it is possible that the *CLM1* and *CLM2* homologs from fusaria other than members of the Sambucinum complex are not involved in CUL biosynthesis.

## 3. Discussion

In this study, purified compounds were used to demonstrate that CUL and DON have a synergistic phytotoxic effect on the economically important plants wheat, barley, and corn (Figure 7). The phytotoxicity was most apparent when the concentration of CUL exceeded that of DON (Figure 1 and Figure 4). This is consistent with the hypothesis that CUL inhibits the glycosylation of DON by serving as a competing alternate substrate for UDP-glucosyltransferases [19].

CUL also had a synergistic phytotoxic affect in combination with 15-ADON, 3-ADON and NX-3, but not with NIV (Figure 5d and Figure 6c). Contrary to expectations, the presence of CUL negated the modest but significant phytotoxicity of NIV observed in hydroponic assays (Figure 6c). The key structural difference between the other trichothecene compounds tested and NIV is that NIV has a C4 hydroxyl-group. Interestingly, the size of the functional group at the C4 position was the limiting factor in the ability of the rice UGT to glycosylate C4-acetylated compounds, such as T-2 toxin. Plant UGTs typically have broad substrate specificity but maintain stringent regioselectivity. Although relatively few UGTs capable of glycosylating DON have been characterized in cereals [22,26,27], UGTs accepting both DON and NIV as substrate have been identified in barley and rice [30,38], and glycosylated NIV has been reported in wheat [39,40]. Therefore, it is unlikely that the results herein are due to a lack of NIV glycosylation in wheat or a NIV-specific UGT that is not inhibited by CUL. If the mechanism of the synergistic phytotoxicity is based on the inhibition of glucosylation, our findings suggest that UGTs may display preferential binding for particular substrates. It is possible that NIV was preferentially detoxified in the presence of CUL, even when CUL was at more than two-fold the concentration of NIV (Figure 6c). This contradicts the competing alternate substrate hypothesis [19].

The use of phytotoxicity assays that allowed for controlled application of known amounts of individual and combined mycotoxins enabled us to visualize the direct effects of each treatment in the absence of the pathogen and any unintended changes to other metabolites. Both the *C. reinhardtii* and plant root growth inhibition assays have previously been used to evaluate the direct phytotoxic effects of trichothecenes [7,26,33], and have similarly demonstrated that DON and other structural analogs are capable of inhibiting plant growth. In contrast to our results, Wang and Miller [5] observed a direct phytotoxic effect of CUL on wheat coleoptile tissues. However, the phytotoxic effect was predominantly observed on tissues treated at 1000 µM CUL, which was approximately 100-fold greater than what was used in the present study (8.4 µM). In the previous study [5], only the most FHB-susceptible wheat variety ‘Concorde’ exhibited a significant inhibition in coleoptile tissue growth when exposed to CUL at a concentration (10 µM) that was comparable to that used in the current study. Additionally, in contrast to mycotoxin phytotoxicity results obtained from coleoptile tissue growth assays, the root elongation assays did not distinguish between FHB-susceptible and moderately resistant wheat varieties (Figure 7a). It is possible that the observed differences between studies are due to variability among wheat varieties or the fact that excised coleoptile tissues are more sensitive to CUL and DON than root tissues.

Although increased phytotoxicity should theoretically favor the aggressiveness of a hemibiotrophic pathogen that derives nutrients from dead or dying plant cells for the latter part of its lifecycle, it has been challenging to conclusively demonstrate that the production of CUL is directly associated with increased pathogen virulence in planta. As an alternative to metabolically engineered *Fusarium* strains, which were reportedly altered in production of other metabolites [14,31] making it difficult to assess the role of CUL in host infection, we attempted to assess the role of CUL in pathogen virulence by evaluating the relationship between the ratio of CUL/DON and the AUDPC from 15 independent isolates of *F. graminearum*. Although the synergistic phytotoxic effects were most apparent when CUL was present at a higher concentration than DON, the CUL/DON ratio and AUDPC were only modestly correlated, and the relationship was negative, indicating that strains that produced more CUL than DON tended to cause less disease (Figure 8a). The CUL/DON ratios reported herein are comparable to those reported in previous publications [27,30,41]. It is possible that the ratio of total CUL + CUL glucoside / DON + D3G would be more closely correlated with disease. However, it is unlikely that the direction of the relationship would change.

Nevertheless, the amount of CUL was positively correlated with disease (AUDPC) (Figure 8b), as was the amount of DON (Figure 8c). However, the sum of both CUL and DON was most closely correlated with disease (Figure 8d), suggesting that the production of both these metabolites contributes to disease.

It is important to also consider DON as the direct source of the phytotoxicity. If CUL contributes to inhibition of DON detoxification, its effect on phytotoxicity is indirect. Of the four spikes with the highest AUDPC values, two had approximately 1000 µg/g of DON (Figure 8 blue points), while the other two spikes only had half the amount of DON (Figure 8 red points), and yet they had similarly high AUDPC values. The spikes with half the amount of DON had CUL/DON ratios of 0.6 and 0.5, while the spikes with high amounts of DON had CUL/DON ratios of 0.2 and 0.14. When the amount of CUL did not exceed that of DON, the production of CUL still appears to benefit disease development. *Fusarium* pathogens may utilize two different strategies to achieve phytotoxicity during disease development: (1) produce large amounts of DON or (2) produce both CUL and DON.

Since CUL and DON are both derived from the same sesquiterpenoid precursor FPP, it is possible that there are trade-offs during biosynthesis. For example, the production of too much CUL could limit DON production. As observed in Figure 8, several wheat spikes were contaminated with primarily CUL, and only minimal amounts of DON. Additionally, these mycotoxin concentrations were quantified at the end of the disease progression assay (21 days post inoculation). During the first 7 days post inoculation, differences in expression of CUL and DON biosynthetic genes suggest that CUL production may be greater than DON production during the first week of infection (Figure 9). Therefore, it is possible that the synergistic phytotoxic effect between CUL and DON in planta relies on the coordinated amount of production of each metabolite at the appropriate time. In light of this, and the involvement of CUL in increasing the phytotoxicity, new control strategies of FHB may include disrupting the coordinated regulation of CUL and DON biosynthesis.

Consistent with the synergistic function of CUL with trichothecenes in *F. graminearum*, the biosynthetic pathways of both metabolites appear to be conserved among members of the Sambucinum complex that are more closely related to *F. graminearum*. CUL production has been confirmed in other DON-producing species, such as *F. boothii,* but also in predominantly NIV-producing species, such as *F. cerealis* and *F. dactylidis* [42,43]. More distantly related members of the Sambucinum complex, *F. sambucinum, F. poae, F. langsethiae, F. sporotrichioides* and *F. longipes,* which typically produce neosolaniol, NIV and diacetoxyscirpenol (DAS), T-2, T-2, and DAS, respectively, lacked one or both CUL biosynthetic genes (Figure 10). Interestingly, the trichothecenes produced by species lacking one or both CUL genes have either a hydroxyl or acetyl group at the C4 position, which may limit the synergistic function of CUL. In this situation, there may be less selective pressure to maintain the CUL biosynthetic pathway. However, homologs of the CUL biosynthetic genes were not restricted to trichothecene-producing fusaria. They were also found in species of the Tricinctum and Burgessii complexes. Additional research is needed to verify if these genes are indeed involved in the biosynthesis of CUL. Mycotoxin analyses of various *F. avenaceum* stains grown on rice for 7 days did not identify any CUL production [43], but some longiborneol was detected, suggesting conserved functionality of *CLM1* [44]. Therefore, it is possible that CUL and/or its biosynthetic intermediate may serve additional unknown functions in *Fusarium*.

## 4. Materials and Methods

### 4.1. Mycotoxins

15-ADON and culmorin were isolated from liquid cultures of *F. graminearum* B4-1 [45]. DON was prepared by quick hydrolysis of 15-ADON with 0.1 N NaOH. 3-ADON was prepared by feeding DON to liquid cultures of a *Saccharomyces cerevisiae* transformant expressing *F. sporotrichioides TRI101* and *TRI12* [46]. 3,15-diANIV was prepared by feeding 15ADON to a *F. verticillioides* transformant expressing *F. sporotrichioides TRI13*. 4-ANIV and 4,15-diANIV were isolated from *F. dactylidis* NRRL29298 [42]. NIV was prepared by hydrolysis of 4-ANIV and 4,15-diANIV with 0.1 N NaOH. NX-2 was isolated from *F. graminearum* NRRL66038 [4]. NX-3 was prepared by hydrolysis of NX-2 with 0.1 N NaOH.

DON, 3-ADON, 15-ADON, NX-3 and CUL were dissolved in 100% acetone. NIV was dissolved in 100% methanol. The acetone and/or methanol concentrations used in each experiment were adjusted so that all treatments received the same solvent combination and concentration, and comparisons were always made to solvent controls, which were conducted in parallel with the treatments.

### 4.2. Phytotoxicity Assays Using Chlamydomonas reinhardtii

The phytotoxicity of *F. graminearum* sesquiterpenoid mycotoxins was evaluated individually and in combination using the model system *C. reinhardtii* as previously described [7]. Briefly, 25 mL flasks containing 10 mL of HSHA (high salt, high acetate) liquid media spiked with known amounts of purified mycotoxins were inoculated with 1 × 10^5^ cell/ mL *C. reinhardtii* strain CC-125 and allowed to grow on an orbital shaker (200 rpm) at room temperature, under 24 h florescent light. On days 3, 4 and 5 post inoculation, the concentration of cells within each flask was estimated using a hemocytometer. Initially, various mycotoxin concentrations were tested to determine the best experimental concentration for further studies.

Significant differences between *C. reinhardtii* growth with the different treatments were determined using an analysis of variance with repeated measures (RM ANOVA) followed by Tukey–Kramer’s honestly significant difference (HSD) test. All treatments were conducted in triplicate and experimentally replicated at least three times.

### 4.3. Root Phytotoxicity Assays

Synergistic phytotoxicity of *F. graminearum* mycotoxins was assessed on wheat, barley and corn. Hard red spring wheat varieties ‘Norm’ and ‘Alsen’ from Minnesota and North Dakota Agricultural Experiment Stations, respectively, were used for the analyses on wheat. ‘Norm’ is susceptible to *Fusarium* head blight, while ‘Alsen’ contains the *Fhb1* and *Fhb5* loci and is considered moderately resistant [33]. FHB resistant barley ‘Robust’ seed was purchased from the local seed distributer Kelly Seed Company in Peoria, IL. Additionally, ‘95SR316A’, a susceptible barley variety, was kindly provided by Tom Baldwin (USDA/ARS). We utilized one dent and one sweet corn variety, “Nothstine Dent”, an heirloom variety, and ‘Silver Queen’, a sweet hybrid. Both varieties were acquired from Jonny’s Selected Seeds. In general, sweet corn is considered to be more susceptible than dent corn.

Seeds were surface sterilized by submersion in 10% bleach for 3 min with gentle shaking, followed by four rinses with sterile water. The surface-sterilized seeds were then pre-germinated on moist filter paper for 24 h prior to being placed on water agar containing the different combinations of mycotoxins.

Root phytotoxicity assays were initially conducted in Nunc^TM^ Square Bioassay Dishes, dimensions 245 × 245 × 25 mm (Thermo Scientific, Waltham, MA, USA), containing 150 mL of 1.5% water agar with mixtures of mycotoxins. After autoclave sterilization of the water agar, but prior to solidification of the agar, known amounts of toxin and solvent controls were added to the agar, which was then poured into the assay plates. The plates were placed at a 10° angle to create a gradient in agar thickness from one side of the plate to the other. After the agar solidified, a straight line, perpendicular to the gradient in agar thickness, was drawn midway across the bottom of the plate. Thirty surface-sterilized pre-germinated seeds were then placed on the agar surface along this line, orienting the radicle toward the thicker agar and the coleoptile toward the thinner agar. For the first two days, the plates were placed under a grow light at a 30° angle, but once the roots had attached to the agar, the angle was increased to 80°. After five days of growth, the root length was measured. Each experiment was conducted as least three times.

However, several roots on the DON and DON plus CUL plate assays appeared to be avoiding direct contact with the mycotoxins by growing out of the media into the humid airspace. Therefore, we developed a hydroponic assay, in which the plant roots were grown directly in the aqueous solution containing the mycotoxins. The hydroponic containers were constructed from empty pipette tip boxes and filled with glass beads to minimize the amount of solution used. Eight to fifteen seeds were placed in each container, and three containers were used per treatment. After five days of growth, the plants were removed from the solution, placed on a flat surface and their root length was measured.

Initially, an ANOVA was performed to determine if there were significant differences between the experimental replicates. If no significant difference was found (*p* > 0.05), the data were combined (*n* = 90). If significant differences were detected (*p* < 0.05), each experimental replicate was analyzed independently (*n* = 30).

For the analysis of difference between ‘Norm’ wheat root length treated with CUL and other sesquiterpenoid mycotoxins, an ANOVA followed by Tukey–Kramer’s HSD test was performed. For the analyses of data including two different varieties, a 2 × 4 (variety × treatment) full factorial ANOVA was used. If variety was not a significant factor, the two varieties were evaluated as one and only a single ANOVA followed by Tukey–Kramer’s HSD test was performed. However, if there were significant differences between the varieties, two independent ANOVAs and Tukey–Kramer’s HSD tests were conducted for each variety.

### 4.4. Wheat Fusarium Head Blight Assays

The relationship between disease severity and the ratio of CUL to DON was evaluated by point inoculating ‘Alsen’ wheat, monitoring disease progression over time, and then plotting the AUDPC against the quantified amount of CUL to DON. Using Microsoft Office Excel, a linear trendline was graphed to visualize the trend of the data and the correlation between the AUDPC and CUL/DON. The coefficient of determination (*R^2^*) was used to assess the degree of the correlation, and the slope of the trendline indicated the direction of the relationship.

In an effort to account for variability in CUL and DON production during disease development, 15 different *F. graminearum* strains were used: 38746, 38762, 38811, 38986, 47571, 52005, 52512, 52955, 06-219, 06-225, 06-270, 13MN1-6, F333, F342, and F344 [47]. ‘Alsen’ plants were grown to anthesis and point inoculated by injecting 10 µL of a single *F. graminearum* strain at 1 × 10^5^ spores/ mL into the third floret from the top counting along one side. The inoculated heads were covered with plastic bags for three days to ensure high relative humidity during initial infection. Three independent heads were inoculated per strain. Thus, a total of 45 heads were evaluated. The number of diseased florets was recorded on days 7, 10, 14, 17 and 21 post inculcation and used to calculate the area under the disease progression curve (AUDPC). At 21 days post inoculation, each head was individually collected, lyophilized, and ground for mycotoxin analysis.

### 4.5. Mycotoxin Extraction and Quantification

Ground material (0.3–0.5 g) was extracted with 10 mL of extraction solvent (acetonitrile-water, 86:14 *v*/*v*) in a 50 mL polypropylene screw cap tube with shaking for 15 min. After centrifugation, 5 mL of extract was purified through a MycoSep 225 Trich cartridge (Romer Labs, Union, MO, USA). A 2 mL aliquot of the purified extract was transferred to a 1-dram vial and dried under a stream of nitrogen. Trimethylsilyl (TMS) derivatives were prepared by adding 100 µL of a 100:1 freshly prepared mixture of N-trimethylsilylimadazole/trimethylchlorosilane (TMSI/TMCS; Sigma-Aldrich, St. Louis, MO, USA) to the dried extract. After 30 min, 900 µL isooctane was added to the reaction mixture followed by 1 mL water. The mixture was agitated gently until the organic (top) layer became translucent. The organic layer was then transferred to 2 mL autosampler vials for gas chromatography–mass spectrometry (GC-MS; Agilent, Santa Clara, CA, USA)) analysis. TMS derivatives of purified DON (0.3125–80 µg) and culmorin (0.3125–80 µg) were similarly prepared with TMSI/TMCS and used to construct standard curves for quantitation.

GC-MS analyses were performed on an Agilent 7890 (Santa Clara, CA, USA) gas chromatograph fitted with an HP-5MS column (30 m, 0.25 mm, 0.25 µm) and a 5977 mass detector. The injection temperature was kept at 250 °C, and the column flow rate was 1 mL/min. A temperature program was used with initial column temperature of 150 °C for 1 min, and then increased to 280 °C at 30 °C min^−1^ and held for 3.5 min. Selective ion monitoring (SIM) was applied to detect the characteristic ions of diTMS-culmorin with a fragment ion (*m/z* value) of 157.1 as the target ion and 183.1, 245.1, 277.1, 292.1 and 367.1 as reference ions, and triTMS-DON with a fragment ion of 235.1 as the target ion and 259.1, 295.1, 392.2, 422.2, and 512.2 as reference ions.

### 4.6. Transcriptional Analysis of CUL and DON Biosynthetic Genes

A FHB-susceptible wheat variety ‘Norm’ was used for dip inoculation and gene expression study, as described in [48]. Briefly, flowering wheat heads were dipped into 1 × 10^5^ /mL macroconidia in 0.02% Tween 20 solution. Six wheat heads were collected and divided into three groups for each time point from day 1 to day 7 post inoculation. The collected wheat heads were frozen in liquid nitrogen, lyophilized, and pulverized. RNA isolation, cDNA synthesis and qPCR assays were performed. Gene expression levels were calculated with 2^−∆∆Ct^ values using cDNA from a 7-day old culture of *F. graminearum* PH-1 grown on V8 as reference. β-tubulin was used as an internal control for normalization of gene expression. Primers for CLM1 (FGSG10397, CLM2 (FGSG_17598)) and β-tubulin were as follows: FGSG_10397-RT-For: 5′ -CGACCAACTCAAACTCACCTAT-3′ and FGSG_10397-RT-Rev: 5′- GAGCCACAAGGGATGTAGAAG-3′; FGSG_17598-RT-For: 5′- GCCGACGATGACATCTACTATG -3′ and FGSG_17598-RT-Rev: 5′- CGGGTCTTGATAGGTTTCTG -3′; FGSG_09530-RT-For: 5′ -CTTCGTCGAGTGGATTCCTAAC-3′ and FGSG_09530-RT-Rev: 5′ -TCCTGGATAGAGGTGGAGTTT-3′.

### 4.7. Statistical Analyses

All statistical analyses were performed using JMP (version 13.1.0, Cary, NC, USA, 2017) statistical software. Individual analyses have been discussed in each section of the methods.

### 4.8. Genomic Analysis of the Distribution of CUL and Trichothecene Biosynthetic Genes

Distribution of CUL and trichothecene biosynthetic genes was assessed using genome sequence data for 53 selected species of *Fusarium* representing 10 of the 22 species complexes that have been described for the genus (Table S1) [35,49]. Genome sequences for non-NRRL strains were downloaded into CLC Genomics Workbench (version 9.5.1, Qiagen, Redwood City, CA, 2016) (hereafter CLC) from databases at the National Center for Biotechnology Information. Genome sequences for NRRL strains were generated using the MiSeq sequencing platform (Illumina, San Diego, CA, USA) and assembled using CLC. DNA libraries for sequencing were constructed from 1 ng of genomic DNA using the Illumina Nextera XT DNA Library Preparation Kit (Illumina, San Diego, CA, USA) following the manufacturer’s specifications. Sequence reads were processed in CLC following the manufacturer’s specifications, except that reads were screened against 120 bacterial genomes prior to processing and assembly; reads with high sequence identity to bacterial sequences were excluded from further processing. The presence and absence of *CLM* and *TRI* genes were assessed using the BLASTn function within CLC, using gene sequences from *F. graminearum* PH-1 as queries [13,14,50,51].

To map the presence and absence of the biosynthetic genes to *Fusarium*, species trees were inferred from nucleotide sequences of 11 primary-metabolism genes that have been used previously for species tree inference in *Fusarium* and other fungi (Table S2) [35,37,52,53]. Coding region sequences of individual genes were aligned using MUSCLE as implemented in the program MEGA (version 7.0.26, 2019) [54]. Two methods were then used to infer species trees from the alignments. First, tree alignments for individual genes were inferred by maximum likelihood analysis as implemented in IQ-Tree (version 1.6.7, 2018) using the ultrafast bootstrap approach [55,56]. The resulting 11 trees were then subjected to extended consensus tree analysis as implemented in RA×ML (version 8.0.20, 2014) [57], and support for branches in the consensus tree was evaluated using the internode certainty approach [58]. Second, alignments of individual primary metabolism genes were concatenated using SequenceMatrix [59], and a tree was inferred from the resulting concatenated alignment by maximum likelihood analysis with ultrafast bootstrapping as implemented in IQ-Tree [55]. Coding region sequences for *CLM1* and *CLM2* were also aligned with MEGA7, and the resulting alignments for each gene were subjected to maximum likelihood analysis with IQ-Tree as described above.

## Supplementary Materials

The following are available online at https://www.mdpi.com/2072-6651/11/10/555/s1, Figure S1: Maximum likelihood trees inferred from coding region sequences of the culmorin biosynthetic genes *CLM1* and *CLM2*, Figure S2: *Fusarium* species shown in Figure 10 but with branch support values, Table S1: Species, strains and genome sequences used in this study, Table S2: List of housekeeping genes to infer *Fusarium* species trees.
