# Supplementary material for: Synergistic Phytotoxic Effects of Culmorin and Trichothecene Mycotoxins
Source: Toxins (Basel). 2019 Sep 20;11(10):555. doi: 10.3390/toxins11100555 (PMC6833022; doi:10.3390/toxins11100555)
Supplement: Supplementary file 1 [file toxins-11-00555-s001.pdf]

toxins

Supplementary Materials: Synergistic Phytotoxic Effects of Culmorin and Trichothecene Mycotoxins

Rebecca Wipfler, Susan P. McCormick, Robert H. Proctor, Jennifer M. Teresi, Guixia Hao, Todd J. Ward, Nancy J. Alexander and Martha M. Vaughan

Table S1. Species, strains and genome sequences used in this study.

Species	Strain Number[a]	NCBI Accession No.
F. acaciae-mearnsii	NRRL 34207	None
F. acuminatum	CS5907	CBMG000000000
F. aethiopicum	NRRL 46738	None
F. algeriense	NRRL 66648	PVPY00000000
F. armeniacum	NRRL 25141	None
F. asiaticum	NRRL 28720	GCA_001717835
F. avenaceum	Fa05001	GCA_000769215
F. aywerte	NRRL 25410	None
F. beomiforme	NRRL 25174	PVQB00000000
F. boothii	NRRL 29105	None
F. brasilicum	NRRL 31281	None
F. camptoceras	NRRL 13381	QGED00000000
F. cerealis	NRRL 25805	None
F. circinatum	FSP 34	AYJV00000000
F. culmorum	UK99	FJUU00000000
F. dactylidis	29380	None
F. equiseti	NRRL 66338	QGEB00000000
F. fujikuroi	IMI58289	GCA_900079805
F. gaditjirrii	NRRL 45417	None
F. graminearum	PH-1	AACM00000000
F. kyushuense	NRRL 25348	None
F. langsethiae	Fl201059	JXCE00000000
F. longipes	NRRL 20695	PXOG00000000
F. louisianense	NRRL 54197	SRX3107725
F. lunulosporum	NRRL 54521	None
F. miscanthi	NRRL 26231	None
F. nepalense	NRRL 54222	None
F. nygamai	MRC8546	LBNR00000000
F. oxysporum	FOSC3a	AFML01000000
F. oxysporum	Fol4287	AAXH00000000.1
F. poae	2516	PVQB00000000
F. praegraminearum	NRRL 39664	LXHY00000000.1
F. proliferatum	NRRL 62905	GCA_900029915
F. pseudograminearum	CS3096	AFNW00000000
F. sambucinum	NRRL 13708	LSRD00000000
F. scirpi	NRRL 66328	QHHJ00000000
F. solani	77-13-4	ACJF00000000
F. sporotrichioides	NRRL 3299	PXOF00000000
F. temperatum	CMWF389	LJGR00000000
F. torreyae	NRRL 54149	None
F. torulosum	NRRL 22747	None
F. tricinctum	NRRL 25481	None
F. udum	F-02845	NIFK00000000
F. venenatum	A3-5	GCA_900007375
F. verticillioides	FGSC 7600	AAIM00000000
FIESC12	NRRL 66336	QHHI00000000
FIESC15	NRRL 31160	QGEA00000000
FIESC23	NRRL 66325	QGDZ00000000
FIESC25	NRRL 66324	QGDY00000000
FIESC28	NRRL 66322	QGDX00000000
FIESC29	NRRL 66334	QHHH00000000
FIESC33	NRRL 66335	QHHG00000000
FIESC33	NRRL 66339	QHKN00000000
FIESC5	CS3069	CBMI00000000
FIESC5	NRRL 66337	QGEC00000000

Strains with NRRL designations are accessioned in the USDA Agriculture Research Service Culture Collection. Origins of other strains are indicated in the genome sequence accessions.

Table S2. List of housekeeping genes used to infer *Fusarium* species trees.

Gene	*F. graminearum* Gene Model	Predicted Protein Product
DPA1	FGSG_05421	DNA Polymerase Alpha Subunit
DPE1	FGSG_12863	DNA Polymerase Epsilon Subunit
FAS1	FGSG_05321	Fatty Acid Synthase Alpha Subunit
LCB2	FGSG_04102	Sphinganine Palmitoyl Transferase Subunit 2
MCM7	FGSG_07105	DNA Replication Licensing Factor
RPB1	FGSG_00916	RNA Polymerase Largest Subunit
RPB2	FGSG_02659	RNA Polymerase 2nd Largest Subunit
TEF1	FGSG_08811	Translation Elongation Factor 1-alpha
TOP1	FGSG_06874	Topoisomerase
TSR1	FGSG_04403	Ribosomal Biogenesis Protein
TUB2	FGSG_09530	Tubulin Beta Subunit

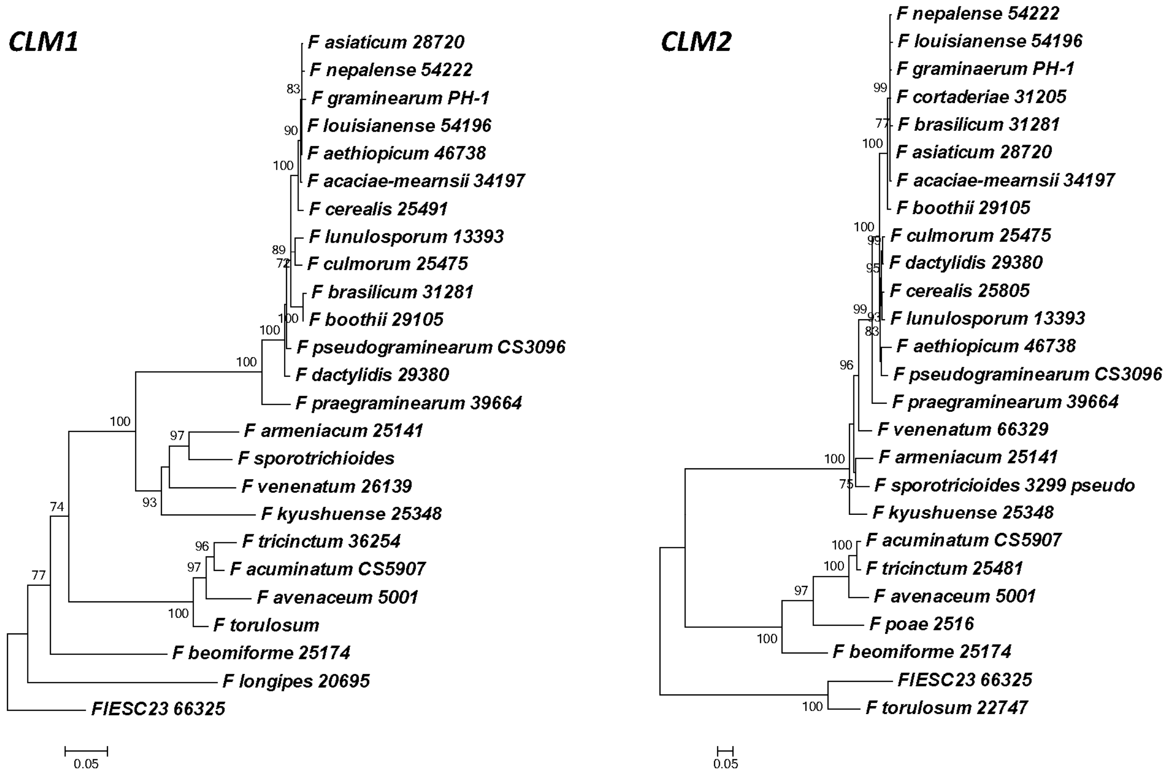

Figure S1. Maximum likelihood trees inferred from coding region sequences of the culmorin biosynthetic genes *CLM1* and *CLM2*. The sequences were aligned using MUSCLE as implemented in the program MEGA [54], and the resulting alignments were subjected to maximum likelihood analysis with ultrafast bootstrapping as implemented in IQ-Tree [1,2].

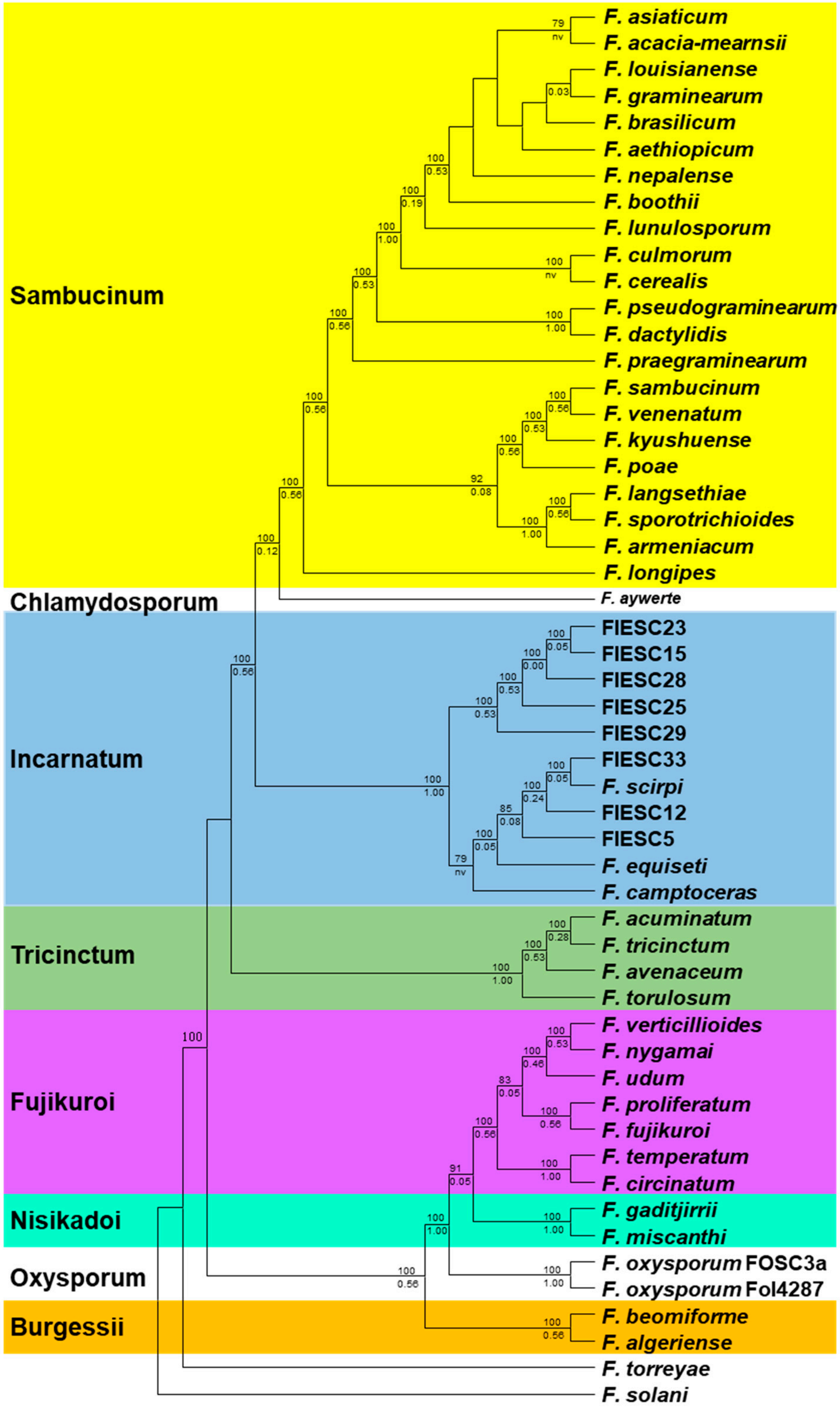

Figure S2. *Fusarium* species shown in Figure 10 but with branch support values. Numbers above branches are bootstrap values based on 1000 pseudoreplicates. Numbers below branches are internode certainty values determined from an extended consensus tree inferred from the 11 maximum likelihood trees inferred for individual primary-metabolism genes [3]. The tree shown in this figure was inferred by maximum likelihood analysis of concatenated alignments of 11 primary-metabolism genes (Table S2). The abbreviation nv (no value) indicates that no internode certainty value was generated for the branch shown in the tree inferred from concatenated sequences. Species complexes are delineated with colored boxes, and the complex names are indicated on the left using the species name after which complexes are named.

References

1. Nguyen, L.T.; Schmidt, H.A.; von Haeseler, A.; Minh, B.Q. IQ-TREE: A fast and effective stochastic algorithm for estimating maximum likelihood phylogenies. *Mol. Biol. Evol*. **2014**, *32*, 268–274.
2. Minh, B.Q.; Nguyen, M.A.; von Haeseler, A. Ultrafast approximation for phylogenetic bootstrap. *Mol. Biol. Evol*. **2013**, *30*, 1188–1195.
3. Kobert, K.; Salichos, L.; Rokas, A.; Stamatakis, A. Computing the internode certainty and related measures from partial gene trees. *Mol. Biol. Evol*. **2016**, *33*, 1606–1617.

© 2019 by the authors. Licensee MDPI, Basel, Switzerland. This article is an open access article distributed under the terms and conditions of the Creative Commons Attribution (CC BY) license (http://creativecommons.org/licenses/by/4.0/).
